# Three doses of prototypic SARS-CoV-2 inactivated vaccine induce cross-protection against its variants of concern

**DOI:** 10.1038/s41392-022-00920-4

**Published:** 2022-02-25

**Authors:** Tianhong Xie, Shuaiyao Lu, Zhanlong He, Hongqi Liu, Junbin Wang, Cong Tang, Ting Yang, Wenhai Yu, Hua Li, Yun Yang, Hao Yang, Lei Yue, Yanan Zhou, Fengmei Yang, Zhiwu Luo, Yanyan Li, Hong Xiang, Yuan Zhao, Jie Wang, Haixuan Wang, Runxiang Long, Dexuan Kuang, Wenjie Tan, Xiaozhong Peng, Qihan Li, Zhongping Xie

**Affiliations:** 1 Institute of Medical Biology, Chinese Academy of Medicine Sciences & Peking Union Medical College, Yunnan Key Laboratory of Vaccine Research and Development on Severe Infectious Diseases, 650118 Kunming, China; 2 The NHC Key Laboratory of Biosafety, National Institute for Viral Disease Control and Prevention, China CDC, 102206 Beijing, China

**Keywords:** Experimental models of disease, Infectious diseases

## Abstract

Variants are globally emerging very quickly following pandemic prototypic SARS-CoV-2. To evaluate the cross-protection of prototypic SARS-CoV-2 vaccine against its variants, we vaccinated rhesus monkeys with three doses of prototypic SARS-CoV-2 inactivated vaccine, followed by challenging with emerging SARS-CoV-2 variants of concern (VOCs). These vaccinated animals produced neutralizing antibodies against Alpha, Beta, Delta, and Omicron variants, although there were certain declinations of geometric mean titer (GMT) as compared with prototypic SARS-CoV-2. Of note, in vivo this prototypic vaccine not only reduced the viral loads in nasal, throat and anal swabs, pulmonary tissues, but also improved the pathological changes in the lung infected by variants of Alpha, Beta, and Delta. In summary, the prototypic SARS-CoV-2 inactivated vaccine in this study protected against VOCs to certain extension, which is of great significance for prevention and control of COVID-19.

## Introduction

SARS-CoV-2 has caused an unprecedented pandemic in the world history, currently with more than 220 million confirmed cases and almost five million deaths. Everyday there are still new cases reported worldwide. SARS-CoV-2 and its disease COVID-19 affect almost everything in the world, including healthcare, education, tourism, aviation, real estate and housing, sports industry, politics, and country relationships.^1^ Given with the experience of SARS-CoV, governors and scientists have paid great attentions to SARS-CoV-2 at the beginning of outbreak. According to the chain of infection (the reservoir of infectious agent, route of transmission and susceptible host), quarantine, wearing masks and environmental disinfection are enforced and efficiently controls the first two steps of the infection chain.

However, vaccination for the susceptible host is widely considered to be the last and best way to control SARS-CoV-2. Theoretically, if a group of 70–80% people get vaccinated and resistant to COVID-19, herd immunity can be established to prevent the remaining small population of unvaccinated people from SARS-CoV-2 infection.^2^ Therefore, vaccines against SARS-CoV-2 have been developing globally via various technical routes, including the traditional inactivated vaccine, recombinant protein vaccine, viral vector vaccine, DNA vaccine, and mRNA vaccine.^3,4^ Until July 2021, there are 322 candidate vaccines proposed in the world, 99 vaccines in clinical studies, 25 at phase III, and 18 under emergency use authorization.^5^ At the time of writing, more than 600 million doses of SARS-CoV-2 vaccines have been given in the world (Our World in Data), covering all people at 12 years or older, and pregnant women.^6^

Vaccination plays critical roles in controlling the transmission of SARS-CoV-2. However, there are some concerns about these rapidly developed and novel vaccines, such as antibody enhancement effect^7–9^ and thrombosis,^10–12^ which are reported to be possibly associated with SARS-CoV-2 vaccines. In particular, SARS-CoV-2 variants are emerging one after another with new characters, including immune escape, more transmission and virulence,^13–15^ which has brought great challenges to vaccine R&D, vaccine users and agency of control and prevention of COVID-19. Nevertheless, vaccine against the prototypic SARS-CoV-2 strain has been still used globally since it is difficult to produce the variant-specific vaccines in such a short time. However, everyone in the world has the same concern about the cross-protection of current vaccines against these emerging variants,^16,17^ although clinical studies show prototypic vaccines are effective in circulating variants, mainly the reduction of COVID-19 severity and mortality based on the WHO definition of vaccine efficacy (>50%).^18^ However, for those clinical studies, it is very hard to evaluate and compare the cross-protection of prototypic vaccines among several variants at one time via the same standard. Non-human primate models of COVID-19 are widely and successfully used in research and development of SARS-CoV-2 vaccines.^19^ Therefore, in this study, we evaluated protection of prototypic SARS-CoV-2 inactivated vaccine against emerging variants of concern (VOCs) in vitro and in vivo to provide guidelines for SARS-CoV-2 vaccination, prevention and control of COVID-19.

## Results

### Prototypic SARS-CoV-2 inactivated vaccine induces cross-neutralizing antibodies against emerging variants of concern

**Fig. 1:**
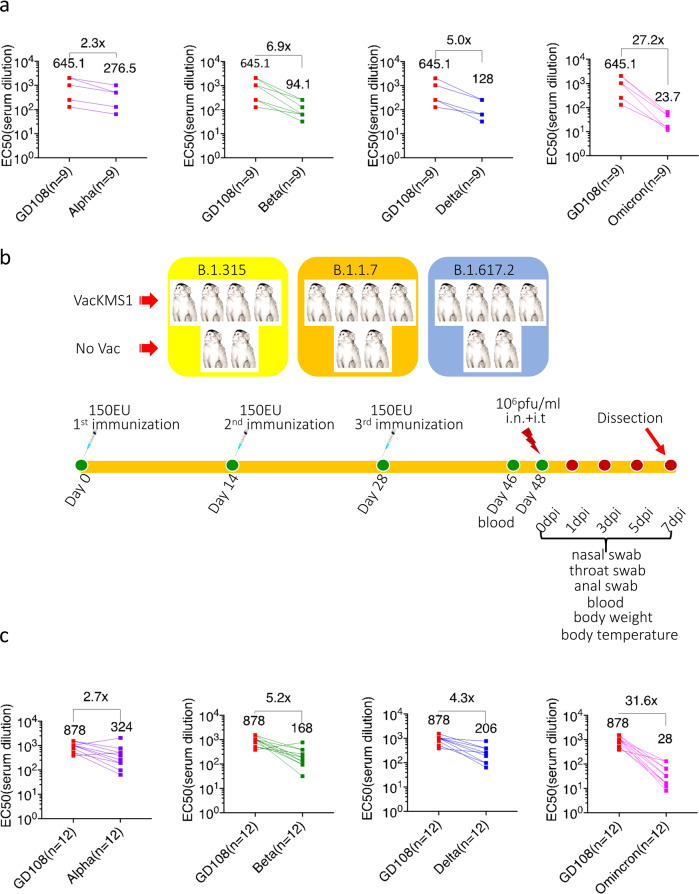
Prototypic SARS-CoV-2 inactivated vaccine induces neutralizing antibodies against its variants of concern (VOCs). **a** Serum samples were collected from vaccinated volunteers by the prototypic SARS-CoV-2 vaccine for micro-neutralization assay (described in materials and methods). Factor change of the prototypic strain GD108 over variants was calculated by GD108 GMT/VOCs GMT. **b** Animal experimental scheme. Sixteen about 1-year-old rhesus monkeys were used in this study. Twelve animals were vaccinated three times with 150 EU prototypic SARS-CoV-2 inactivated vaccine (VacKMS1) as indicated. At 20 days post the third immunization, animals were inoculated with 1 × 10^6^ pfu/ml variants via the intranasal and intratracheal route. Animals were monitored and samples were collected on 0dpi, 1dpi, 3dpi, 5dpi and 7dpi. All animals were euthanized and dissected on 7dpi. **c** Serum samples were collected from vaccinated rhesus monkeys by the prototypic SARS-CoV-2 vaccine for micro-neutralization assay (described in materials and methods). Factor change of the prototypic strain GD108 over variants was calculated by GD108 GMT/VOCs GMT

In order to evaluate effectiveness of prototypic SARS-CoV-2 vaccine against its VOCs, cross-neutralization assay was performed on clinical serum samples from volunteers vaccinated with three doses of prototypic SARS-CoV-2 inactivated vaccine KMS-1 (VacKMS1) against the emerging VOCs, Alpha (B.1.1.7), Beta (B.1.351), Delta (B.1.617.2), and Omicron (B.1.1.592). Strikingly, vaccinated volunteers produced antibodies that could neutralize all tested variants with neutralizing titers up to 2048, although geometric mean titer (GMT) of neutralizing antibody against VOCs were lower, compared with the prototypic SARS-CoV-2 (Fig. 1a). In order to confirm these clinical results, we vaccinated 12 rhesus monkeys with three doses of this inactivated vaccine (Fig. 1b). At 18 days post 3rd vaccination, serum samples were collected for further analysis. Micro-neutralization assay revealed that vaccinated rhesus monkeys produced neutralizing antibody against prototypic viruses with titers of 512-1024 (GMT = 878), against the Beta variant B.1.351 with titers of 32–768 (GMT = 168), Alpha variant B.1.1.7 with titers of 64–2048 (GMT = 324), Delta variant B.1.617.2 with titers of 64–768 (GMT = 206), and Omicron variant B.1.1.592 with titers of 8–128. The factor changes of four variants GMT over that of prototypic virus were 5.2, 2.7, 4.3 and 31.6 respectively (Fig. 1c), which was consistent with the clinical results from vaccinated volunteers (Fig. 1a).

**Fig. 2:**
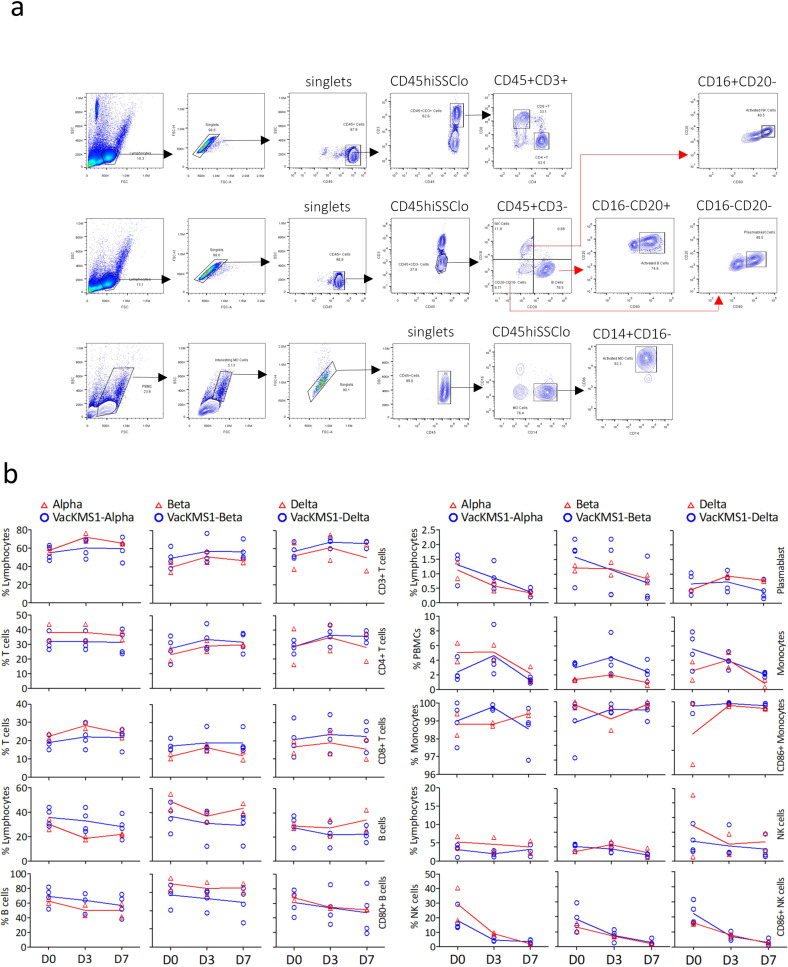
Cellular immune responses induced by vaccination of the prototypic SARS-CoV-2 inactivated vaccine in rhesus monkey. **a** The representative dot and contour plots showed populations of total T cells, CD4 + T cells, CD8 + T cells, B cells and monocytes from a rhesus macaque. Firstly, singlets were gated to eliminate doublets and then gating was performed on the indicated subsets of lymphocytes. Then combination of cell type-specific markers was used to define immune cells, such as CD45 + CD3 + for total T cells that were further differentiated by CD4 and CD8 markers, CD45 + CD3-CD16-CD20 + to determine total B cells and CD45 + CD3-CD16-CD20- for NK cells with activation marker CD80, CD45 + CD14 + CD16- for monocytes with activation marker CD86. **b** Mean frequencies of each subsets of immune cells are shown as curve plots (each plot represents one animal with the red line for unvaccinated animals and the blue line for vaccinated animals)

Dynamic analysis of cellular immune responses was conducted in the peripheral blood monocytes (PBMCs) of rhesus monkeys via flow cytometry. The results indicated that an increased proportion of T cells and monocytes occurred on 3 dpi, followed by slight decrease in Beta (B.1.351) or Delta (B1.617.2)-infected animals, in which vaccination of prototypic SARS-CoV-2 could restore the proportion of T cells. Whereas, frequencies of B cells and NK cells decreased upon variants infection, which was recovered by vaccination only in Alpha (B.1.1.7)-infection animals (Fig. 2). These results suggested that the prototypic SARS-CoV-2 inactivated vaccine could induce the immune protection against the emerging SARS-CoV-2 variants.

### Vaccination of the prototypic SARS-CoV-2 inactivated vaccine reduces the shedding of SARS-CoV-2 variants

**Fig. 3:**
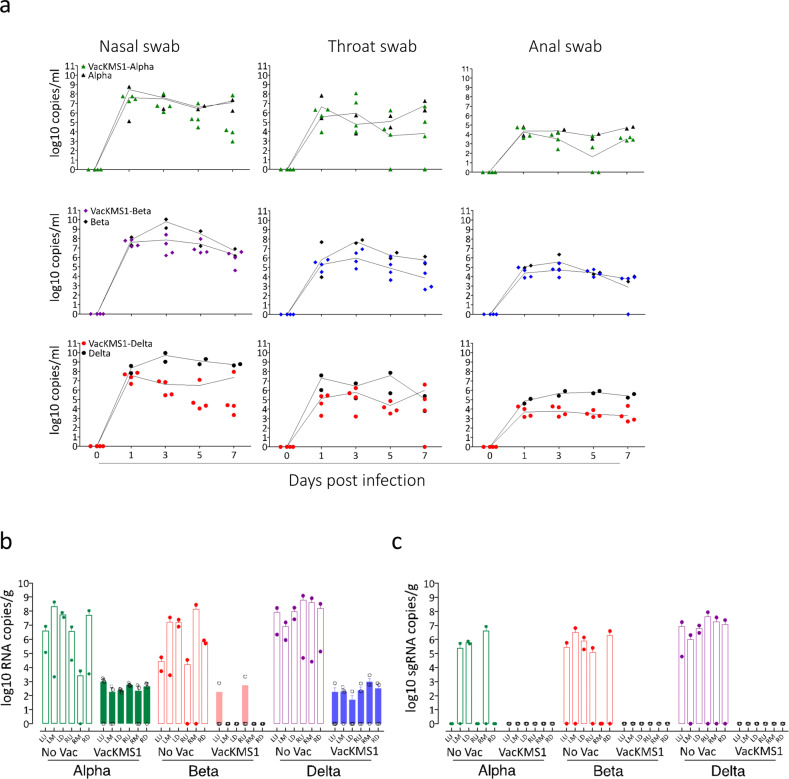
SARS-CoV-2 inactivated vaccine inhibited the shedding of VOCs. **a** Viral loads in swab samples collected at the indicated time points were determined via qRT-PCR as described in the materials and methods section. Six lobes of each lung were harvested on 7 dpi for analysis of viral genomes (**b**) and subgenomes (**c**) via qRT-PCR to evaluate the replications of VOCs in pulmonary tissues. LU upper lobe of left lung, LM middle lobe of left lung, LD down lobe of left lung, RU upper lobe of right lung, RM middle lobe of right lung, RD down lobe of right lung

Next, we evaluated the cross-protection of prototypic SARS-CoV-2 vaccine against its variants in rhesus monkeys. Vaccinated animals were challenged with SARS-CoV-2 variants at 20 days post 3rd immunization. We found relatively lower copy number of viral RNA in nasal swabs, throat swabs and anal swabs from vaccinated animals as compared to those from unvaccinated animals post inoculations of VOCs, suggesting that vaccination could reduce the shedding of viral variants to some extent, particularly for the variant Delta (B.1.672.2) (Fig. 3a). On the day 7 post infection (dpi), lower copy numbers of viral genomes and subgenomes were observed in the lung tissues of vaccinated animals, indicating vaccination could inhibit the replication of SARS-CoV-2 variants in lung (Fig. 3b, c). These results demonstrated that neutralizing antibody induced by prototypic SARS-CoV-2 inactivated vaccine could reduce the transmission and replication of emerging VOCs.

### The prototypic SARS-CoV-2 inactivated vaccine ameliorates the pathological changes in the lung caused by infections of SARS-CoV-2 variants

**Fig. 4:**
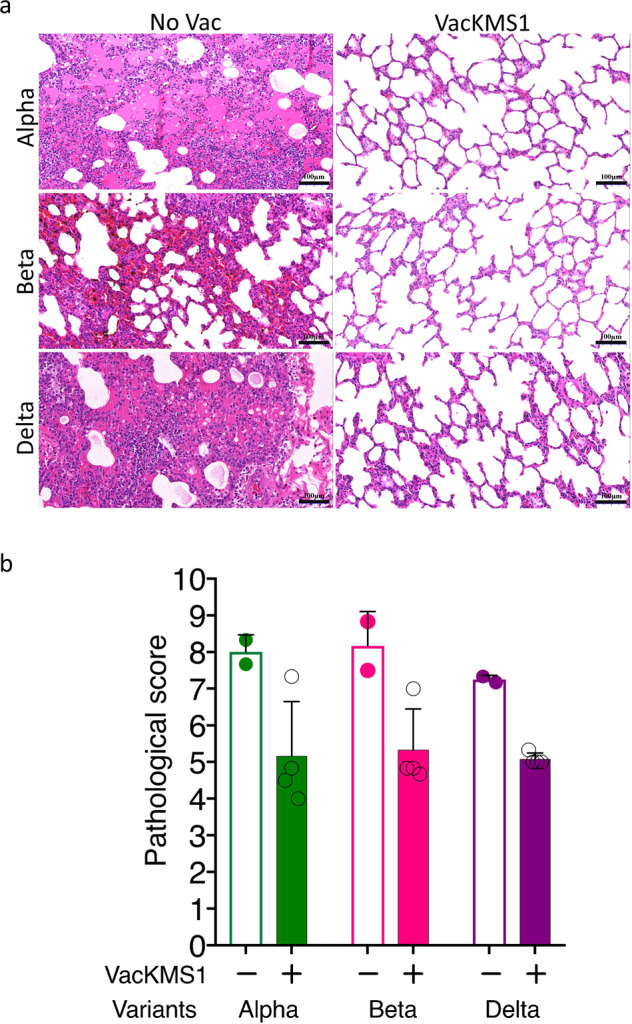
Vaccination of prototypic SARS-CoV-2 inactivated vaccine mitigated the pathological changes in the pulmonary tissues of VOCs infected animals. Rhesus monkeys (vaccinated or unvaccinated) were inoculated i.n/i.t with 1 ml of 10^6^ pfu respective variants as indicated. On 7 dpi, all animals were euthanized and dissected. Lung tissues were harvested and prepared for H&E staining and further histological analysis by experienced pathologists (**a**). According to the severity of histological lesion, pathological score was given to each tissue sample (**b**). The pathological changes were obviously alleviated in the vaccinated animals infected with variants

**Fig. 5:**
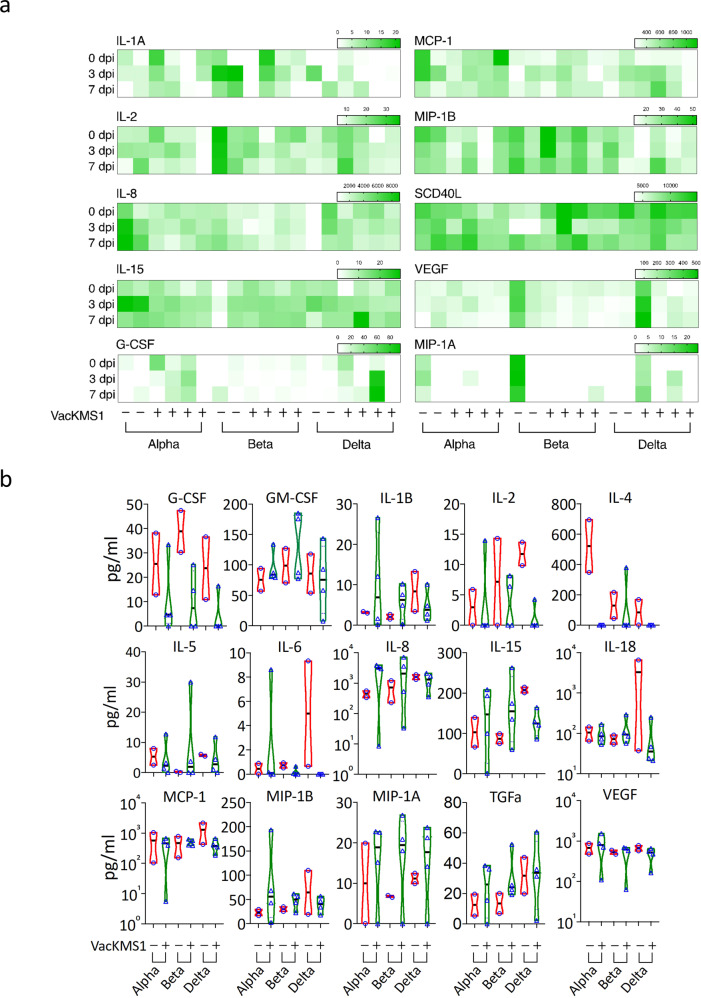
Effects of the prototypic SARS-CoV-2 vaccine on inflammatory responses in VOC-infected animals. Inflammatory cytokines in serum (**a**) and lung tissue (**b**) samples from unvaccinated or vaccinated animals were measured by Luminex multiplex assays as described in the “materials and methods” section. All detectable cytokines were shown here

To further evaluate the cross-protection induced by inactivated vaccine against variants, we examined the histopathological changes in lung tissues harvested on 7 dpi since pulmonary pathology is widely accepted to be the golden standard for COVID-19. Typical histopathological lesions were observed in the lung tissues from all vaccinated or unvaccinated animals post challenge of viral variants, including thickening of pulmonary septum, pulmonary septal hemorrhage, infiltration of Inflammatory cells, thickening of local vascular wall, lymphocytic nodule, vascular lumen thrombosis, dispersed dust cells, pigmentation, proliferation of pulmonary epithelial cells, cellular or proteinaceous exudate (Fig. 4a), which was similar to those in lungs of prototypical SARS-CoV-2 challenged animals. Strikingly, it is promising that vaccination of prototypic inactivated vaccine improved the pathological changes in variants-challenged groups (Fig. 4b). To understand the protective mechanism, we evaluated the inflammatory cytokines in vaccinated or unvaccinated animals. In serum samples, levels of inflammatory cytokines didn’t correlate with the vaccination or viral inoculation (Fig. 5a). Notably, vaccination declined the concentration of multiple inflammatory cytokines in lung tissues from the delta strain Delta (B.1.617.2) infected animals, but not the other two variants (Fig. 5b). Together, these results indicated that the prototypic SARS-CoV-2 vaccine provided certain cross-protection against the circulating VOCs.

## Discussion

Although the SARS-CoV-2 vaccines have been used on an unprecedented scale worldwide,^20^ which plays a key role in combating COVID-19,^21^ the cross protection of vaccine against emerging variants is being a global concern.^16,22,23^ This is exacerbated with frequently reported breakthrough infection of SARS-CoV-2 variants in vaccinated population.^24^ Promisingly, there are some clinical studies showing that prototypic SARS-CoV-2 vaccine could provide some degree of protection against emerging variants, mainly reduce the severity and mortality of COVID-19.^25–27^ This study, via in vitro neutralization assay and in vivo rhesus monkey model, demonstrated that prototypic inactivated vaccine induced neutralizing antibody and further provided the cross-protection of animals against circulating VOCs, including the decreased virus shedding, replication and improved pathological changes in lung. These results are similar with those reported in the clinical studies.^28,29^

Level of neutralizing antibody reflects host’s resistance to viral infection and may be a predictor of vaccine efficiency,^30^.^31^ Results of clinical samples from in vitro neutralization assay of SARS-CoV-2 shows that neutralizing antibody induced by prototypic vaccines can crossly neutralize VOCs to some extension.^32^ Analysis of large scale of clinical data suggests that neutralizing antibody levels in patients could be highly related with immune protection from symptomatic COVID-19.^33,34^ However, it is hard to determine the optimal level of neutralizing antibody that could provide protection against SARS-CoV-2, particularly VOCs. In this study, neutralizing assay via authentic viruses suggests that neutralizing antibody induced by prototypic SARS-CoV-2 had decreasing ability of neutralization against variants, titers of which were from 1:8 to 1:2048 (Fig. 1c), but these antibodies showed certain protection crossly against emerging Alpha, Beta and Delta variants in vivo (Fig. 4). For the variant Omicron, it could be still neutralized in vitro by prototypic SARS-CoV-2 induced antibody although NT decreased (Fig. 1a–c). Since neutralizing antibody titres can be used as predictors of protection against SARS-CoV-2 variants,^33,35^ we predict prototypic SARS-CoV-2 vaccine could provide cross-protection against the Omicron variant in vivo. Furthermore, the optimal level of neutralizing antibody should be determined for cross-protection against SARS-CoV-2 variants, which will be valuable to guide the use of vaccines.

Due to medical ethics, there are some common limitations for most of clinical studies about COVID-19, which makes it hard to accurately compare the cross protection of prototypic SARS-CoV-2 vaccine against its variants. In contrast, advantages of the present study are: firstly, the established NHP model that is widely accepted to be suitable for COVID-19 researches; secondly, animals could be immunized with the same batch of vaccine at the same dose; thirdly, the equal amount of viral particles was used to infect the vaccinated animals; lastly, the viral shedding, replication, inflammatory cytokines and immune response could be evaluated at different time points to dynamically explore the cross protection and possible mechanism of vaccine immunity. Therefore, the highlight of this study is the analysis of the cross-protections among variants comparatively in both longitudinal and sectional approaches, which is very hard to be performed in clinical patients.

In unvaccinated rhesus monkeys that were inoculated with SARS-CoV-2 variants, we observed the viral shedding from nasal swabs, anal swab and throat swabs, immune responses, viral replication and classical pathology in in lung tissues, which is similar to the reported NHP models of COVID-19,^19,36^ indicating the success of NHP models of SARS-CoV-2 variants infection. However, the shortcoming of this study is that only two animals were used in each unvaccinated group due to the limitation of non-human primate resource. Therefore, we could not conduct the statistical analysis to show the significant differences between vaccinated and unvaccinated groups.

In conclusion, the results of this study suggests that the prototypic SARS-CoV-2 inactivated vaccine could induce certain protections against the circulating VOCs, although further experiments are urgently anticipated to elucidate the mechanism of this cross-protection.

## Materials and methods

### Ethics and biosafety statement

Collection of human serum sample was approved by the institutional review board and performed after inform consent signed. All animal procedures were approved by the Institutional Animal Care and Use Committee of the Institute of Medical Biology, Chinese Academy of Medical Science (ethics number: DWLL202106 003), and performed in strict accordance with the guidelines for the National Care and Use of Animals approved by the National Animal Research Authority (PR China) and the

Experimental Animal Ethics Committee of the Institute in the ABSL-4 facility of the National Kunming High-level Biosafety Primate Research Center, Yunnan, China.

### Vaccines

The prototypic strain of SARS-CoV-2 KMS-1 was inactivated and developed for vaccine by Medical Biology (IMB), Chinese Academy of Medical Sciences (CAMS), as described previously.^37^ This vaccine is named as VacKMS1 in this study for convenience. The concentration of vaccine antigen was determined by enzyme-linked immunosorbent assay (ELISA) and defined as ELISA unit (EU).

### Viruses

In addition to the prototypic strain of SARS-CoV-2, four emerging VOCs were chosen for this study. The prototypic SARS-CoV-2 strain (GD108) and Beta variant (B.1.351, GDPCC-nCOV84, CSTR.16698.06. NPRC 2.062100001) were gifted from Guangdong CDC, Alpha variant (B.1.1.7, SARS-CoV-2/C-Tan-BJ202101(B1.1.7), CSTR.16698.06. NPRC 2.062100002) from China CDC, Delta variant (B.1.617.2, CQ79, CSTR.16698.06. NPRC 6. CCPM-B-V-049-2105-8) from Chongqing CDC and Omicron variant (B.1.1.529 CCPM-B-V-049-2112-18) from Institute of laboratory animal sciences, CAMS&PUMC. All strains of viruses were amplified in Vero-E6 or Vero cells, cultured in DMEM supplemented with 10% FBS and 1% P/S. The titration of virus for challenging was determined via the plaque assay as previously described.^19^ The whole genomes of GD108, Alpha, Beta, Delta and Omicron were sequenced (data not shown) and characteristic mutation sites in S gene were analyzed (Supplementary Fig. 1).

### Animals

The non-human primates in this study were rhesus monkeys that were all male and around 1-year-old. Twelve animals were immunized with 150 EU of the prototypic SARS-CoV-2 inactivated vaccine three times at 0, 14 and 28 days, followed by the intranasal and intratracheal (0.5 ml each) inoculation of three VOCs respectively (four animals for each variant) at the dosage of 10^6^ pfu/animal/1 ml at 20 days post the third immunization (Fig. 1b).

### Determination of viral loads and viral replications

Viral loads were determined as described previously.^19^ Briefly, total RNA was purified via ZYMO kit according to the protocol recommended by manufacture. Real-time RT-PCR was used to quantify the viral RNA genome or subgenome using TaqMan Fast Virus 1-Step Master Mix (Thermo Fisher Scientific, USA) and purified SARS-CoV-2 RNA as a standard curve on a CFX384 Touch Real-Time PCR Detection System (Bio-Rad, USA).

### Micro-neutralization assay

Serum was serially diluted by two-fold with DMEM medium in 96-well plates for micro-neutralization assay. Each well contained 120 ul of each diluted serum sample. Viruses were diluted to the concentration of 2000 pfu/ml with DMEM. 120 μl of diluted viruses was added to each well containing equal volume of diluted serum sample, followed by incubation at 37 °C and 5% CO2 for 60 min. Meanwhile, 100 ul of Vero-E6 cells (2 × 10^5^ cells/ml) were seeded into each well of 96-well plate. After 1 hour incubation, 100 ul of the mixture of serum and viruses in each well were transferred to Vero-E6 cells in each well of 96-well plate. Plates were incubated at 37 °C and 5% CO2 for 5 days. Cytopathogenic effect was recorded to evaluate the neutralizing ability of serum sample. The TCID50 at the highest dilution was defined as the neutralization titer (NT). Factor change of NT was calculated by GMT of prototypic SARS-CoV-2 over variants GMT.

### Cytokine assay

The concentration of cytokine in serum and tissue samples was determined via the MILLIPLEX MAP Non-Human Primate Cytokine Magnetic Bead Panel—Immunology Multiplex Assay (Cat# PRCYTOMAG-40K, Millipore, USA) according to the manufacturer’s protocol on a Bio-Plex machine (Bio-Rad, USA). The 23 inflammatory cytokines in this panel are IL-1ra, IL-1β, IL-2, IL-4, IL-5, IL-6, IL-8/CXCL8, IL-10, IL-12p40, IL-13, IL-15, IL-17A/CTLA8, IL-18, G-CSF, GM-CSF, IFN-γ, MCP-1/CCL2, MIP-1α/CCL3, MIP-1β/CCL4, sCD40L, TGF-α, TNF-α, and VEGF.

### Flow cytometric analysis of peripheral blood monocytes

Peripheral blood monocytes (PBMCs) were prepared from anticoagulant whole blood via Ficoll-Paque^TM^ Plus (Cat#17144003, cytiva, Sweden). 5 ml of peripheral blood was collected in a vacutainer blood collection tube containing anticoagulant. Equal volume of PBS was added to dilute the blood. This diluted blood was transferred and slowly dropped onto 5 ml Ficoll-Paque^TM^ Plus, followed by centrifugation at room temperature (RT) and 2000 rpm for 20 min with the highest acceleration and the lowest deceleration. PBMCs at the thin and white 2nd layer was harvested and transferred to a 15 ml corning tube. PBMCs were washed twice by addition of PBS and centrifugation at RT and 2000 *rpm* for 5 min, stained with fluorescent-conjugated antibodies against cell-specific markers and analyzed on the flow cytometer Cytoflex (Beckman).

### Histology

On 7 dpi, animals were euthanized and dissected for histopathological analysis. Six lobes of each lung (three from left and three from right) were harvested for preparing formalin-fixed paraffin-embedded sections (5 μm) and further for hematoxylin and eosin (H&E) staining. The H&E stained slides were evaluated by an experienced pathologist in a double-blind manner. Six-grade scoring system from the least severe to the most severe (0–5) was used to evaluate the histopathological changes in every slide based on the severity of histopathological changes as follows, thickening of pulmonary septum, pulmonary septal hemorrhage, infiltration of Inflammatory cells, thickening of local vascular wall, lymphocytic nodule, vascular lumen thrombosis, dispersed dust cells, pigmentation, proliferation of pulmonary epithelial cells, cellular or proteinaceous exudate. At least five fields in a slide were randomly selected from each lobe of lung tissue of rhesus monkey (upper left, middle left, lower left, upper right, middle right and lower right, a total of six lobes). The average of pathological scores of all lobes is defined as the comprehensive pathological score of the whole lung of the rhesus monkey.

## Supplementary information

Supplementary Materials
