# Supplementary material for: Three doses of prototypic SARS-CoV-2 inactivated vaccine induce cross-protection against its variants of concern
Source: Signal Transduct Target Ther. 2022 Feb 25;7:61. doi: 10.1038/s41392-022-00920-4 (PMC8873345; doi:10.1038/s41392-022-00920-4)
Supplement: Supplementary file 1 — Supplementary Materials [file 41392_2022_920_MOESM1_ESM.docx]

**Supplementary Materials for**

**Three doses of prototypic SARS-CoV-2 inactivated vaccine induce cross-protection against its variants of concern**

Tianhong Xie^1#^, Shuaiyao Lu^1#^, Zhanlong He^1#^, Hongqi Liu^1#^, Junbin Wang^1#^, Cong Tang^1#^, Ting Yang^1^, Wenhai Yu^1^, Hua Li^1^, Yun Yang^1^, Hao Yang^1^, Lei Yue^1^, Yanan Zhou^1^, Fengmei Yang^1^, Zhiwu Luo^1^, Yanyan Li^1^, Hong Xiang^1^, Yuan Zhao^1^, Jie Wang^1^, Haixuan Wang^1^, Runxiang Long^1^, Dexuan Kuang^1^, Wenjie Tan^2*^, Xiaozhong Peng^1*^, Qihan Li^1*^, Zhongping Xie^1*^

1. Institute of Medical Biology, Chinese Academy of Medicine Sciences & Peking Union Medical College, Yunnan Key Laboratory of Vaccine Research and Development on Severe Infectious Diseases, Kunming 650118, China.

2. The NHC Key Laboratory of Biosafety, National Institute for Viral Disease Control and Prevention, China CDC, Beijing 102206, China

This PDF file includes:

Supplementary Figure 1.


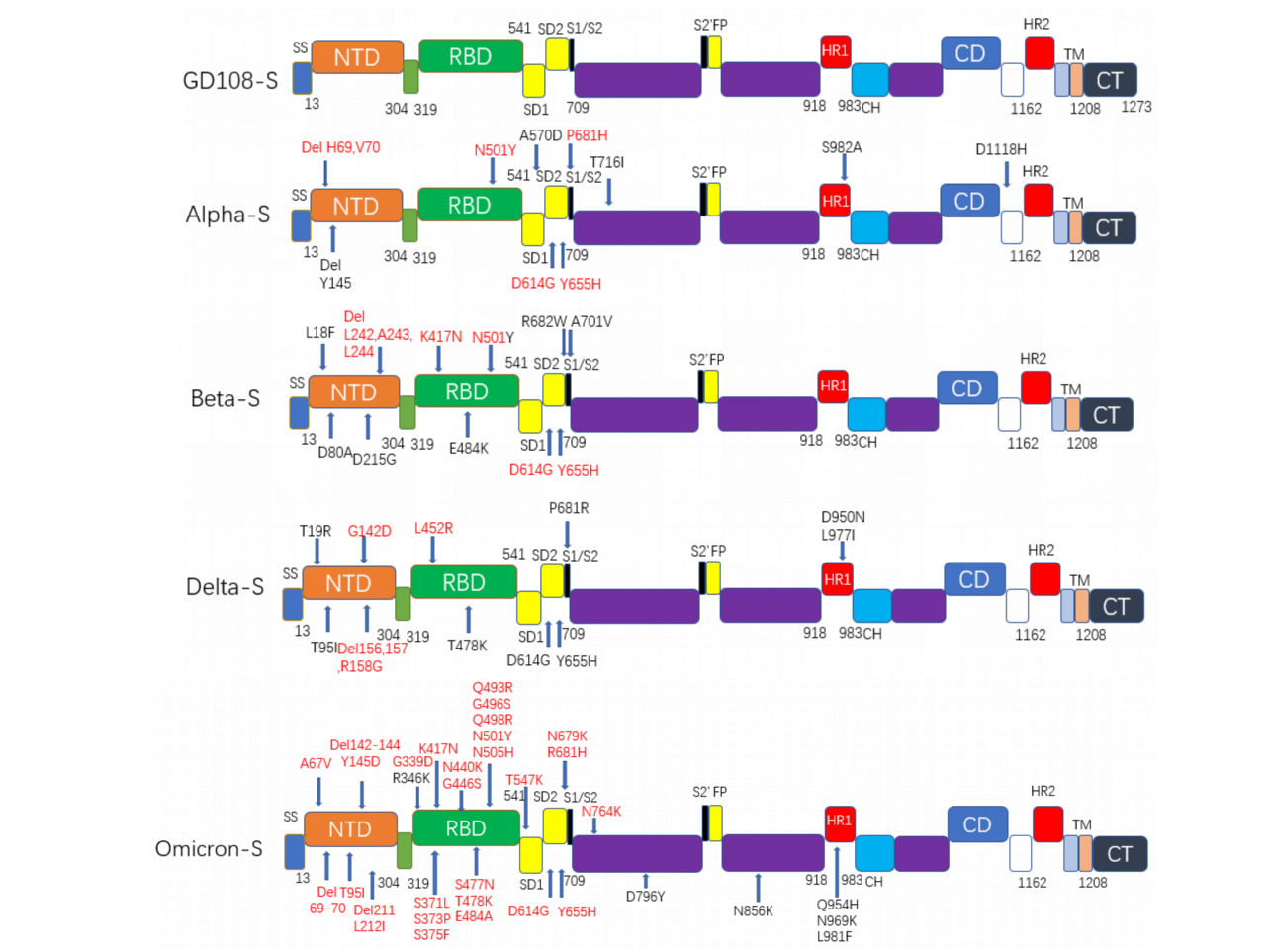


Figure S1. Amino acid sequences of spike protein obtained in this study. Functional domains are boxed in various colors and characteristic mutation sites are labeled in red.
